# The Effects of the Creator’s Situation on Creativity Evaluation: The Rater’s Cognitive Empathy and Affective Empathy Matter in Rating Creative Works

**DOI:** 10.3390/jintelligence10040075

**Published:** 2022-09-26

**Authors:** Yilai Pei, Jiantao Han, Jingwen Zhao, Mengrong Liu, Weiguo Pang

**Affiliations:** 1 Institute of Developmental and Educational Psychology, School of Psychology and Cognitive Science, East China Normal University, Shanghai 200062, China; 2 School of Educational Science, Anhui Normal University, Wuhu 241000, China; 3 Counseling and Support Services, Shanghai Jiao Tong University, Shanghai 200030, China; 4 School of Education, Zhejiang International Studies University, Hangzhou 310023, China

**Keywords:** creative evaluation, cognitive empathy, affective empathy, sympathy

## Abstract

Successful intelligence theory suggests that creativity is necessary for personal achievement outside of intelligence. Unlike intelligence, creativity can develop in a supportive environment. People should consider the situation of disadvantaged groups, which are characterized by low personal achievement and a bad growth environment in creativity evaluation from a caring perspective. This study focuses on the effect of the creator’s situation on creative evaluation and the role of the rater’s empathy (i.e., cognitive empathy and affective empathy) and sympathy in creative evaluation. Four pairs of creator’s situations (by age, physical state, family situation, and economic state) were designed to represent people with disadvantages or advantages. A between-subject design was used with 590 undergraduate students randomly assigned to eight sub-conditions. The participants were asked to assess three products in eight situations. The rater’s empathy and sympathy in creativity evaluation were explored in the overall disadvantage (N = 300) and advantage (N = 290) conditions. The results showed that the participants only provided significantly higher ratings to the creative product made by a child. Cognitive empathy only predicted a creative rating under disadvantaged conditions, and affective empathy negatively moderated this effect. Affective empathy only predicted a creative rating under advantage conditions, and cognitive empathy positively moderated this effect. Affective empathy only predicted a creative rating under advantage conditions, and cognitive empathy positively moderated this effect. The possible mechanisms of the effect and implications for the establishment of a supportive environment for creativity and creativity teaching practice were discussed.

## 1. Introduction

Successful intelligence theory suggests that personal achievement requires both intelligence and creativity (Sternberg 1999). In contrast to intelligence, which is regarded as a stable personal trait, creativity can be developed to a significant extent in a supportive environment (van der Zanden et al. 2020). Given that creativity contributes to personal achievement in various domains (Diedrich et al. 2018) and personal creativity progresses under others’ feedback (Beghetto and Kaufman 2014), there is value in more thoroughly examining the establishment of a supportive environment for creativity.

This study focuses on the question of how people assess a product created by a disadvantaged group. Research shows that disadvantaged groups usually have inadequate creativity development. For example, people who grew up in disadvantaged families tended to have less stimulating home environments, insufficient parental involvement, and experienced maladaptation at school, resulting in inadequate personal development (Rothe et al. 2014; Seabra-Santos et al. 2021; Skopek et al. 2017). Due to inadequate development, the creative performance of people in disadvantaged situations cannot be compared with people in advantageous situations (Xu and Pang 2020). Runco (1993) proposed that creative potential is widely distributed to both disadvantaged groups and advantaged groups and that the low creative performance of disadvantaged groups is related to them having more undiscovered creative potential.

Researchers have discussed ways to protect disadvantaged groups from inadequate development and have devised several related interventions (Archambault et al. 2020; Seabra-Santos et al. 2018). The literature shows that one effective protective factor of inadequate development is the teacher’s positive behavior (e.g., specific praise and encouragement). Specifically, Seabra-Santos et al. (2021) found that teachers give more specific praises when they follow a training program that both promotes the students’ development and reduces the effect of student’s socio-economic disparity. Similarly, van der Zanden et al. (2020) summarized that encouraging adolescents to express their ideas is beneficial to their creativity development. The development of creativity depends on the interactions between daily creative actions and feedback from social circumstances, with communication around the creative product between the creator and the rater being the primary mechanism (Glăveanu 2013). Various pieces of research suggest that people need their ideas and creative works to be understood and accepted because this helps maintain their self-efficacy and intrinsic motivation, which are key factors in creative task performance and creativity development (Amabile 1996; Beghetto and Kaufman 2014; Deci and Ryan 2008; Karwowski and Beghetto 2019; Karwowski and Kaufman 2017; Nease et al. 1999; Oettingen et al. 2012; Yuan et al. 2019).

Empirical studies have demonstrated the role of positive feedback in one’s intrinsic motivation, emotion, and creative performance. Fodor (1990) found that people show more intrinsic motivation and better performance in an engineering problem after receiving positive feedback after the pilot task. Moreover, de Rooij et al. (2017) found that people’s emotions and creativity are influenced by the pattern of subsequent feedback (i.e., four subsequently permutations of positive feedback and negative feedback); the results showed that people who receive positive feedback following negative feedback in the pilot task report significantly higher expectations, more feelings of satisfaction and frustration and have better performance in alternative use tasks (i.e., originality of ideas) than people in the other three conditions. Furthermore, Hon et al. (2013) found that employees under challenge-related stress report the most creative behavior when they perceive more positive task feedback from supervisors, and employees suffering from hindrance stressors are more creative at work when they receive less negative and more positive feedback.

Therefore, both theoretical and some empirical evidence support that others’ positive attitude toward the creator is beneficial to the latter’s creativity development and creative performance. Notwithstanding, whether and how people give more positive evaluations to the products created by the individuals from disadvantaged groups are questions that remain to be answered. Scholars have demonstrated perspective-taking as the mechanism underlying the effect of the creator’s information on interpersonal creativity evaluation (Han et al. 2017). Perspective-taking has a strong connection to empathy, which is defined as the ability to know what the other person is thinking and feeling and to care for one another (Stueber 2017; Vossen et al. 2015). Perspective-taking is also used to evoke empathy (Batson et al. 1997a), with various studies exploring the empathic process, including its underlying cognitive and affective mechanisms, when people see others in disadvantaged situations (Batson 1987; Batson et al. 1997a; Eisenberg et al. 1989). Therefore, this study aimed to investigate the relationship between the creator’s situation, the rater’s empathy, and creative evaluation.

### 1.1. Creator’s Situation and Creative Evaluation

Past studies have generally focused on specific persons who are capable of evaluating creative products with accuracy (Kaufman and Baer 2012; Kaufman et al. 2013). However, judgment accuracy is not always the most important thing for the raters of creative products. For example, in the field of education, the primary task of the teacher is to establish a creativity-supportive learning environment (Beghetto and Kaufman 2014; Patston et al. 2021). Meanwhile, assessments made by teachers in the classroom environment are meant to foster the student’s development, so teachers should generally use formative rather than summative forms of evaluation (Bennett 2011; Black and Wiliam 1998; Bloom 1969; McManus 2008). For example, if a student made a sketchy replication of “The Scream,” the teacher should encourage the student’s creative behavior and put forward constructive proposals, not simply judge whether the product is original enough or not.

In the current study, to distinguish from the typical product-oriented evaluation—which evaluates the product’s originality and effectiveness (Runco and Jaeger 2012)—we define the concept of a creator-development-oriented evaluation as follows: the assessment process which aims to promote the creator’s development. In product-orientated evaluation, the creator’s information is regarded as a source of rating bias (Cheng 2018; Lebuda and Karwowski 2013), but it is essential in creator-development-orientated evaluation. Empirical research has demonstrated the influence of the creator’s information on creative evaluation. For example, the creators’ age, gender, and name can influence the rater’s evaluation (Han et al. 2017; Hennessey 1994; Lebuda and Karwowski 2013). Some other studies found interactions between the creator’s information and the rater’s traits. The gender and ethnicity of raters had interaction effects with the creator’s gender and ethnicity even when the demographic information of the creator was concealed from the raters (Kaufman et al. 2010). (Cheng 2018) also found interaction effects between the rater’s neuroticism and the creator’s dominance and between the rater’s neuroticism and the creator’s friendliness rating bias. One other recent study found that the cultural background of the creator can also affect the creative evaluation, with Russians judging their compatriots significantly higher than the Emirati judged their compatriots in a creative evaluation task (Kharkhurin and Yagolkovskiy 2021) while other studies suggest that people from different cultures, such as Chinese and Americans (Niu and Sternberg 2001) and Chinese and Germans (Tang et al. 2018), have high consensus to the products created by people from different countries.

Some researchers explore the mechanism of the effect of the creator’s information. Han et al. (2017) showed that perspective-taking is what determines the effect of the creator’s information on the rater’s creative evaluation; people put themselves into the creator’s situation and adjust their rating. The concept of a disadvantaged group is relative to that of an advantaged group, and it involves one’s life experience, bodily health and integrity, affiliation, and economic status, which together may cause the loss of control over one’s environment (Kong et al. 2014; Wolff and De-Shalit 2007).

In this study, we focus on four typical, real-life disadvantaged groups: children, people with prolonged illness/disability, orphans, and the economically disadvantaged. Children have a far lower personal development level than adults in physical and cognitive ability, which is related to their mental vulnerability and low resilience (Engle et al. 1996). Moreover, people with prolonged illness and disability have fewer opportunities for personal development (e.g., higher education) and are more susceptible to mental disorders (Moriña 2017; Verhaak et al. 2005). Orphans have lower social support and enrollment and a higher risk of mental health issues than persons with large families (Case et al. 2004; Makame et al. 2002). The economically disadvantaged have lower socioeconomic status than the economically advantaged, and they are characterized by low rates of college enrollment and increased mental health problems (Kong et al. 2014; Wang et al. 2011).

Hence, the four types of disadvantage groups explored in this research are all characterized by lagging personal development and vulnerable mental states. Because disadvantaged groups would likely perform better if they had access to the same favorable conditions as advantaged groups, during the creative evaluation, the raters may give disadvantaged groups higher ratings to reflect the concept of outcome justice (Skitka et al. 2003). Specifically, research shows that people become satisfied with products that meet their expectations (Cardozo 1965). Considering that disadvantaged groups are frequently stereotyped as having a low academic ability, being lazy, and having little interest in improving themselves (Bullock 1999; Cozzarelli et al. 2001; Woods et al. 2005), raters may create lower expectations for the creators from such groups compared to the creators from advantaged groups, potentially leading to greater satisfaction with the first’s creative outputs.

A positive attitude is another factor through which the creator’s situation could influence creative evaluation. Research shows that people generate positive attitudes when they know that the other is in a disadvantaged situation. For example, Batson et al. (Batson et al. 1997a) found that the participants who listened to an interview of a young woman who is in serious need reported higher positive attitudes toward the woman than the group being asked to remain objective to the situation. Decety and Chaminade (2003) found that people viewed the actor who told a sad story with a sad expression to be more likable than either the actor telling a natural story or the actor telling the same sad story with a natural expression. A meta-analysis also demonstrated that people showed more forgiveness for people who are at a disadvantage (Fehr et al. 2010).

In conclusion, we believe that people would change their creativity rating for people who are disadvantaged (i.e., using creator-development-orientated evaluation) based on both cognitive and affective processes.

### 1.2. Empathy, Sympathy, Creator’s Situation, and Creative Evaluation

The concept of empathy is usually described through the empathic process evoked by perceiving another person’s distress (Batson 1987; Batson et al. 1997a; Eisenberg et al. 1989), it can be split into its cognitive and emotional components, and sympathy is independent of empathy (Vossen et al. 2015). Cognitive empathy is the ability to comprehend/understand another person’s emotion (Hogan 1969). Affective empathy is the ability to experience another person’s emotion (Mehrabian and Epstein 1972), and sympathy is defined as feeling concern or sorrow for another person’s distress (Clark 2010).

Empathy is the ability to know what the other person is thinking and feeling and caring for one another (Stueber 2017). It is usually referred to as an empathic process evoked by another’s distress Empathy can be split into cognitive and emotional components, and sympathy is relatively independent. Both affective empathy and sympathy are emotional responses to the distress of others, and their difference lies in emotion congruence: the emotion in affective empathy is similar to that of another person, while the emotion in sympathy (i.e., being concerned) differs from that of the other individual (Vossen et al. 2015).

The literature provides conflicting evidence on the relationships between creativity, empathy, and sympathy. At the trait level, and to the best of our knowledge, almost no study reported the direct relationship between trait empathy, sympathy, and divergent thinking. Additionally, few studies reported a positive relationship between empathy and creative activities (Carlozzi et al. 1995; Dostál et al. 2017). Sven and Christian (2018) found that empathy has a positive correlation with creative achievement and an inverted-U relationship with everyday creativity. Yang and Yang (2016) found that the originality of ideas in both divergent thinking and content-specific task (i.e., design a room for an older person) increases when sympathy is induced by showing images of older adults with disadvantage (e.g., ill, economically disadvantaged, hungry, etc.), and that trait affective empathy moderates this effect. However, Salminen et al. (2021) found no effect of state empathy (i.e., evoking by reading the story of a person with a disadvantage) on problem detection (i.e., identifying and listing as many problems that the person in the story may face as possible) and idea generation (i.e., list as many solutions that could help the person in the story as possible).

In addition to these ambiguous findings on the association of creativity, empathy, and sympathy, the role of empathy and sympathy in creative evaluation remains overlooked by scientific scholars. Cognitive empathy has a positive correlation to perspective-taking (Vossen et al. 2015), which is, in turn, beneficial for eliciting, among raters, rating adjustments that take into account the creator’s information (Han et al. 2017). In addition, the cognitive component is at the beginning stage of an empathic process; for example, upon perceiving that someone has a disadvantage, first, one could put oneself in the other’s situation first (i.e., cognitive empathy) and then experience feelings such as warmth, softheartedness, tenderness, compassion, and feel moved by the situation (i.e., sympathy) (Batson et al. 1997a). As a result of such feelings toward another with a disadvantage, one could then desire to console the other, try to make them feel better, or at least hear the other out (Maibom 2017). Therefore, the empathic process contributes to various altruistic behaviors, including helping, sharing, donating, and being concerned for others (Eisenberg et al. 2010).

Moreover, researchers have shown that while cognitive empathy positively associates with perspective-taking and prosocial behavior, the associations with affective empathy are relatively weak in comparison (Vossen et al. 2015; Y. Wang et al. 2017). Meanwhile, sympathy correlates highly with empathic concern, moderately with prosocial behavior, and has a negative relationship with aggressive behavior, whereas neither cognitive nor affective empathy are related to aggressive behavior (Salavera and Usán 2020; Vossen et al. 2015; Y. Wang et al. 2017). Affective responses include feelings of sympathy, compassion, tenderness, and likeness for the other is the core process in generating a positive attitude and further altruistic motivation toward the person with a disadvantage (Batson et al. 2015; Eisenberg et al. 1989). The study by Navarro-Mateu et al. (2019) found that the teacher’s overall empathy (i.e., the aggregate score of four affective empathy items and four cognitive empathy items) is related to positive attitudes toward disadvantaged groups. Thus, people with high affective empathy or high sympathy may be more likely to hold a more positive attitude toward people with a disadvantage, which may result in them providing higher scores to the creative work of people with a disadvantage as a way to practice prosocial behavior.

### 1.3. The Present Study

This study aimed to explore the effect of the creator’s situation and the rater’s empathy (i.e., cognitive empathy and affective empathy) and sympathy on creative evaluation. We hypothesized two different pathways for how the creator’s situation influences creative evaluation. First, the raters may adjust their evaluations based on the creator’s situation at the cognitive level. People evaluate creative work by measuring the creator’s knowledge, life experience, and thinking style by taking the creator’s perspective (Han et al. 2017). Based on prior research (McLoyd 1998; Seabra-Santos et al. 2021), the four typical disadvantaged groups which we explore in this study are all characterized by lagging personal development and vulnerable mental states. Then, because of the rater’s potential low expectations toward disadvantaged groups (Bullock 1999; Cozzarelli et al. 2001; Woods et al. 2005), raters may be more likely to provide higher satisfaction rates toward products created by disadvantaged groups as an expectation effect (Cardozo 1965). In addition, disadvantaged groups could show better performance in creativity-related tasks if they had the same favorable conditions as those in the advantaged groups. Raters may provide higher ratings by following the concept of outcome justice (Skitka et al. 2003).

Second, the raters may adjust their evaluations based on the creator’s situation at the emotional level. People may experience emotions such as compassion, concern, and tenderness when they see people at a disadvantage (Van Lange 2008), as well as may hold a positive attitude toward disadvantaged groups (Batson et al. 1997a). Therefore, using four paired categories (age: child vs. adult; physical state: prolonged illness vs. health; family situation: orphan vs. big family; economic state: economically disadvantaged vs. economically advantaged), we expected higher ratings for products when raters know the creator is at a disadvantage compared to when they know the creator is at an advantage. Accordingly, the first hypothesis is as follows:

**Hypothesis** **1** **(H1).** *Raters provide higher ratings for creative works when they know the creator is at a disadvantage than when they know the creator is at an advantage across the four paired categories.*

Research shows that higher cognitive empathy correlates with better perspective-taking (Vossen et al. 2015). Putting oneself into another’s situation help people understand other’s situations and notice the diversity and consideration of justice (Segal 2011). Hence, we expected a positive relationship between cognitive empathy and creative evaluation when raters know the creator is at a disadvantage but not when rates know the creator is at an advantage. This led to the following hypothesis:

**Hypothesis** **2** **(H2).** *The raters’ cognitive empathy and the creator’s situation interact to influence creative evaluation, with cognitive empathy predicting ratings only when raters know that the creator is at a disadvantage.*

Affective empathy is essential for experiencing emotions similar to that which others are experiencing, and sympathy is the tendency to feel concern or sorrow for others’ distress (Vossen et al. 2015). Moreover, both affective empathy and sympathy contribute to the affective path that leads raters to have more positive attitudes toward the creative products of disadvantaged groups (Batson et al. 1997a; Eisenberg et al. 2010; Fehr et al. 2010).

**Hypothesis** **3** **(H3).** *The raters’ affective empathy and the creator’s situation interact to influence creative evaluation, with affective empathy only predicting ratings when raters know that the creator is at a disadvantage.*

**Hypothesis** **4** **(H4).** *The raters’ sympathy and the creator’s situation interact to influence creative evaluation, with sympathy only predicting ratings when raters know that the creator is at a disadvantage.*

As described before, a rater’s cognitive empathy, affective empathy, and sympathy can influence creative evaluation through two potential paths, and the cognitive component plays a basic and initial function in the empathy process. Accordingly, we expected the positive moderation effect of cognitive empathy on the two relationships of creative evaluation (i.e., affective empathy and sympathy) when raters know that the creator is at a disadvantage. This led to the last two study hypotheses:

**Hypothesis** **5** **(H5).** *The raters’ cognitive empathy positively moderates the effect of affective empathy on creative evaluation when raters know that the creator is at a disadvantage.*

**Hypothesis** **6** **(H6).** *The raters’ cognitive empathy positively moderates the effect of sympathy on creative evaluation when raters know that the creator is at a disadvantage.*

**Figure 1:**
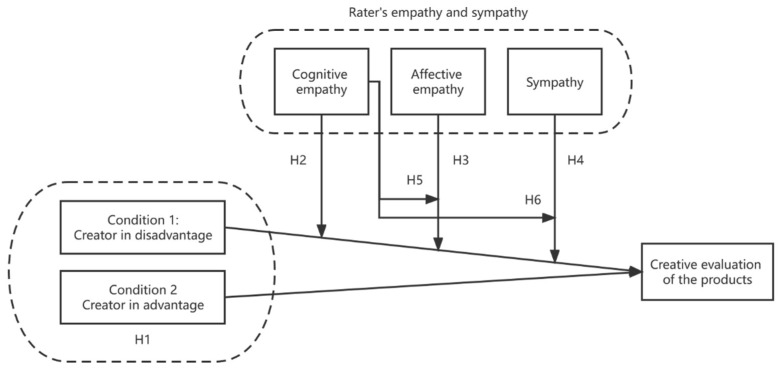
The hypotheses between the creator’s situation, rater’s empathy and sympathy, and creative product evaluation.

All hypotheses in this study are shown in Figure 1.

## 2. Methods

### 2.1. Participants

In total, 590 undergraduate students (age: 19.9 ± 2.3 years, ranging from 17 to 27 years old) from Eastern China Normal University (*n* = 255), Central China Normal University (*n* = 155), and Hefei Normal University (*n* = 180) participated in this study. Following the methods from various prior studies (Dollinger and Shafran 2005; Kaufman et al. 2009, 2005), we treated the recruited undergraduate students as novice raters. There were 300 (121 men and 179 women) and 290 (118 men and 172 women) participants in the disadvantaged and advantaged conditions, respectively. The disadvantaged condition contained four sub-conditions: child (*n* = 70), prolonged illness (*n* = 77), orphan (*n* = 82) and economically disadvantaged (*n* = 71). The advantaged condition contained four sub-conditions: adult (*n* = 74), healthy (*n* = 70), big family (*n* = 79) and economically advantaged (*n* = 67). None of the students had any background in creativity research, and all were recruited and completed the task online. 

The Ethics Committee of East China Normal University approved the protocols of this research (HR: 172-2020); furthermore, written informed consent was obtained from the participants prior to the study onset, and the methods were carried out following the approved guidelines.

### 2.2. Assessment Materials

Three different products with middle-level creativity were used as assessment materials, which were generated through the figure completion (FC) task, alternative use task, and alien stories. These three creative product formats were employed because they are described as being the most frequently used in assessing creative ability (Han et al. 2017; Long 2014). One moderately original idea from the alternative use task (i.e., thinking of the uses of newspaper as much as possible) and one alien story with a novelty score in the middle level were chosen from the study of Han et al. (2017). The painting was chosen from paintings generated using figure completion (FC) tasks in another study (L. Wang et al. 2017). The FC task was designed to test figural divergent thinking, requiring the participants to draw original pictures based on incomplete figures, each completed within 3 min. The participants’ performances on the FC task were assessed by three trained and unbiased raters based on the consensual assessment technique (CAT; Amabile 1982) for originality and elaboration. The originality and elaboration scores were rated on a 5-point scale (1 = *not at all original/elaborate* to 5 = *highly original/elaborate*), and these two scores for the two FC tasks were averaged for every participant. The internal consistency of the originality and elaboration scores were satisfactory (Cronbach’s α coefficients of .72 and .86, respectively). The chosen painting was in the middle level (*originality* = 3.2, *elaboration* = 3.2). All three products are in the middle level of creativity because we aimed to test the effect of the creator’s situation. 

### 2.3. Experimental Design

All of the undergraduate raters were randomly assigned to two conditions (creator at a disadvantage vs. creator at an advantage) with four types (age: child vs. adult; physical state: prolonged illness vs. healthy; family situation: orphan vs. big family; economic state: economically disadvantaged vs. economically advantaged). The disadvantaged group was designed based on categories of life, bodily health and integrity, affiliation, and control over one’s environment (Wolff and De-Shalit 2007). In the disadvantaged condition, participants were informed that the creator has one type of disadvantage (i.e., child, orphan, prolonged illness, or economically disadvantaged). In the advantaged condition, the participants were informed that the creator has one type of advantage (i.e., adult, big family, healthy, or economically advantaged). Following the method proposed by Han et al. (2022), the participants were asked to take the creator’s perspective, including the creator’s level of knowledge, life experience, and thinking style, for 30 s to ensure sufficient processing of the creator’s situation. This operation was performed to ensure the sufficient processing of the creator’s information since we collected data online. After that, the participants were asked to rate the three products on a 1–7 Likert scale (1 = *not creative at all*; 7 = *very creative*), which are labeled with the information of the creator (e.g., “Wang Ming is an orphan, lives helplessly and is friendless. Please rate the creative level of Wang Ming’s products.” All of the introductions are shown in Appendix A). 

### 2.4. Empathy Measures and Controlled Variables

The raters’ trait empathy (cognitive and affective empathy) was measured using the Adolescent Measure of Empathy and Sympathy (Vossen et al. 2015). It contains four items to measure affective empathy (e.g., “I feel scared when a friend is afraid;” *Cronbach’s α* = 0.69), and four items for cognitive empathy (e.g., “I can tell when a friend angry even if he/she tries to hide it;” *Cronbach’s α* = 0.77), and four items for sympathy (e.g., “I feel sorry for someone who is treated unfairly;” *Cronbach’s α* = 0.75). The participants received the following instructions for completing this scale: “We are going to ask you some questions about what you are like and how you normally behave. For each statement, please indicate how often this occurs.” The items were rated on a 5-point response scale ranging from 1–5 (1 = *never*; 5 = *always*). 

Self-assessed creativity was measured by the 23-item Runco Ideational Behavior Scale (RIBS) (Runco et al. 2001). Some example items are as follows: “I have many wild ideas” and “I come up with an idea or solution other people have never thought of.” The scale is responded on a 5-point scale ranging from 1–5 (1 = *never*, 5 = *always*; *Cronbach’s α* = 0.77). 

The correlation between cognitive empathy and affective empathy was 0.467 (*p* < .001), between cognitive empathy and sympathy, was 0.368 (*p* < .001), and between affective empathy and sympathy was 0.437 (*p* < .001). Hence, the relationships between cognitive empathy, affective empathy, and sympathy were at a moderate level, corroborating the results of prior research (Salavera and Usán 2020; Vossen et al. 2015; Y. Wang et al. 2017). 

### 2.5. Data Analysis

All of the analyses were performed using the R package “bruceR,” (version 0.8.5, Bao, https://CRAN.R-project.org/package=bruceR (accessed on 10 September 2022)) in R (version 4.02, R Core Team, Vienna, Austria, (R Core Team 2016)). Simple slope and mediation analysis were implemented by bruceR, which has a strict corresponding relation with Hayes’s SPSS PROCESS macro (Bao 2022; Hayes 2017). The rating scores of the three evaluation materials (painting: *mean ± standard deviation* [SD] = 4.10 ± 1.22; story: *mean ± SD* = 3.83 ± 1.31; idea: *mean ± SD* = 3.85 ± 1.28) were transformed into z-scores, and the average z-score of materials was used for the following analysis. 

Statistical analyses were performed for fixed effects, main effects, and interaction effects; considering a medium effect size of 0.25, an alpha level of 0.05, a number of groups of 2, and a number of covariates of 16, the statistical power was shown to be > 0.99, which is considered excellent (Cohen 2013).

## 3. Results

### 3.1. Effect of Creator’s Situation

The descriptive statistics of the creativity product ratings in the two conditions are presented in Table 1. Analysis of covariance was used to explore how the creator’s situation, cognitive empathy, affective empathy, and sympathy contribute to the creative evaluation. The results showed that the main effect of the creator’s situation (*F* (1, 572) = 18.828, *p* < .001, *η_p_*^2^ = .019) and the RIBS (*F* (1, 572) = 8.807, *p* = .003, *η_p_*^2^ = .022) were significant, while that of sympathy was only marginally significant (*F* (1, 572) = 3.410, *p* = .065, *η_p_*^2^ = .005). There was no significant effect of gender (*F* (1, 572) = 0.385, *p* = .535, *η_p_*^2^ < .001), cognitive empathy (*F* (1, 572) = 1.774, *p* = .183, *η_p_*^2^ = .012), nor affective empathy (*F* (1, 572) = 0.419, *p* = .517, *η_p_*^2^ < .001). See Table 2.

Considering the four pairs of situations (age: child vs. adult; physical state: prolonged illness vs. healthy; family situation: orphan vs. big family; economic state: economically disadvantaged vs. economically advantaged; Table 3), the effect of the creator’s situation only appeared for the child condition. Specifically, the participants gave significantly higher scores to the child than in the other seven conditions (*p* < .003; *Cohen’s d* > 0.50). The participants showed the tendency to give higher scores for four disadvantaged conditions compared with their paired advantaged conditions, but the differences between each pair did not reach statistical significance. The results were as follows: prolonged illness vs. healthy (*p* = .304, *Cohen’s d* = 0.19); orphan vs. big family (*p* = .543, *Cohen’s d* = 0.10); economically disadvantaged vs. economically advantaged (*p* = .560, *Cohen’s d* = 0.10).

### 3.2. Interaction Effect of Rater’s Empathy/Sympathy and Creator’s Situation

**Figure 2:**
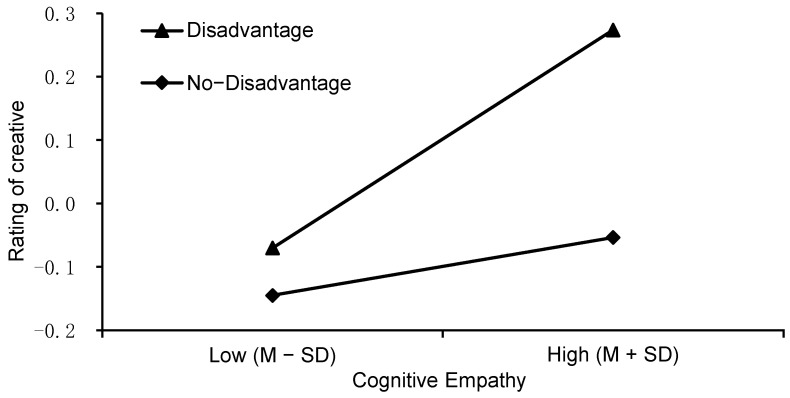
The interaction effect of cognitive empathy and the creator’s situation on creative evaluation.

**Figure 3:**
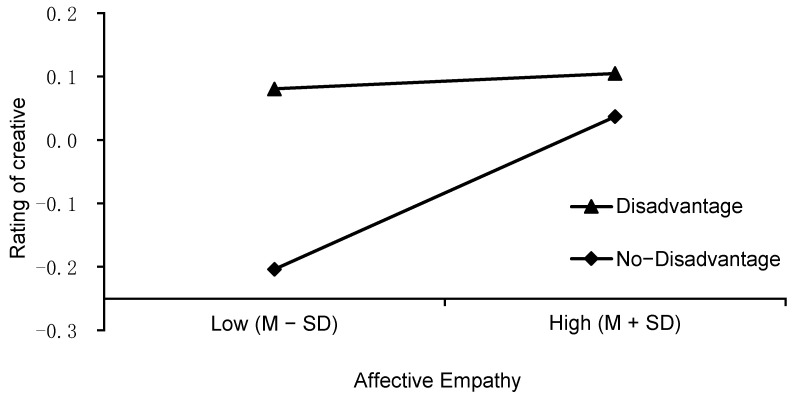
The interaction effect of affective empathy and the creator’s situation on creative evaluation.

We also observed two significant interactions for the creator’s situation: that with cognitive empathy (*F* (1, 572) = 8.080, *p* = .005, *η_p_*^2^ = .006) and with affective empathy (*F* (1, 572) = 6.545, *p* = .011, *η_p_*^2^ = .017). There was a triple interaction between the creator’s situation, cognitive empathy, and affective empathy (*F* (1, 572) = 4.486, *p* = .035, *η_p_*^2^ = .025). The simple effect analysis showed that cognitive empathy (see Figure 2) significantly predicted creative evaluation when raters knew that the creator was at a disadvantage (*β* = 0.279, *t* = 3.04, *p* = .002) but not when they knew that the creator was at an advantage (*β* = −0.100, *t* = −1.03, *p* = .304). In addition, affective empathy (see Figure 3) significantly predicted creative evaluation when the raters knew that the creator was at an advantage (*β* = 0.198, *t* = 2.20, *p* = .028) but not when they knew that the creator was at a disadvantage (*β* = −0.12, *t* = −1.37, *p* = .172).

**Figure 4:**
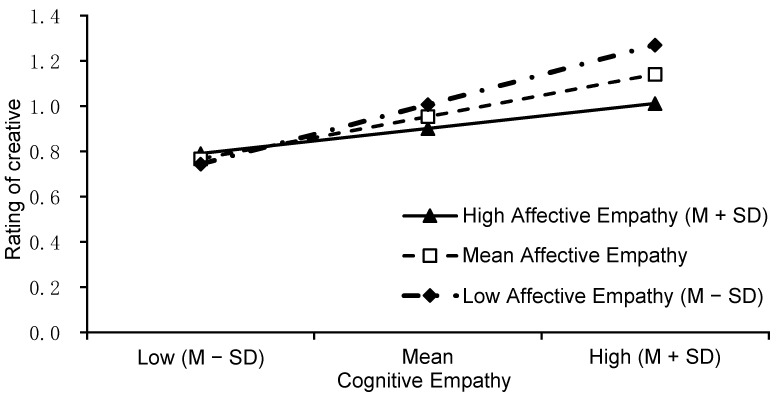
The simple slope of cognitive empathy in different affective empathy levels for the disadvantaged condition.

To explore the triple interaction effect of the creator’s situation, cognitive empathy, and affective empathy, two moderation analyses were implemented in the disadvantaged and advantaged conditions. The results showed that, in the disadvantaged condition, the interaction effect of cognitive empathy and affective empathy was significant (*β* = −0.265, *p* = .017). Simple slope analysis showed that the raters’ cognitive empathy significantly predicted their creative evaluation when their affective empathy was high (*β* = 0.503, *t* = 4.928, *p* < .001) and moderate (*β* = 0.357, *t* = 4.073, *p* < .001). When their affective empathy was low, this prediction only reached marginal statistical significance (*β* = 0.211, *t* = 1.904, *p* = .058). See Figure 4. 

**Figure 5:**
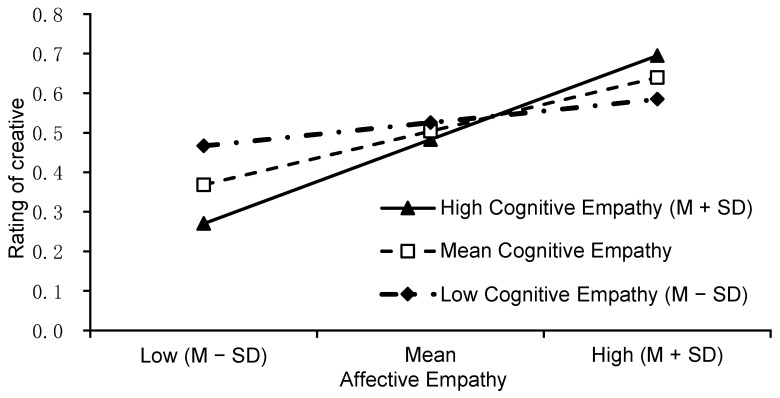
The simple slope of affective empathy in different cognitive empathy levels for the advantaged condition.

In the advantaged condition, the interaction effect of cognitive empathy and affective empathy was significant (*β* = 0.236, *p* = .003). Simple slope analysis showed that the raters’ affective empathy predicted their creative evaluation when their cognitive empathy was high (*β* = 0.352, *t* = 3.951, *p* < .001) and moderate (*β* = 0.225, *t* = 2.853, *p* = .005), but not when it was low (*β* = 0.098, *t* = 1.090, *p* = .277). See Figure 5.

## 4. Discussion

This study aimed to examine the effects of the creator’s situation on creative evaluation and the role of the raters’ empathy and sympathy in creative evaluation. We hypothesized that people rate creative works higher from disadvantaged groups than those from advantaged groups (H1). This is partially supported, as the effect was significant only in the age condition (child vs. adult). In the other three situation pairs (prolonged illness vs. healthy; orphan vs. big family; economically disadvantaged vs. economically advantaged), the participants only showed a tendency toward providing higher rates when they knew the creator was at a disadvantage, with the related results showing low effect sizes (0.09 < *Cohen’s d* < 0.19) and no statistical significance. The result for the age condition was consistent with the findings in previous studies (Han et al. 2017; Hennessey 1994), but it does not support the general assumption of the effects of the creator’s disadvantaged situation on creative evaluation. One possible explanation is the group differences between participants (i.e., undergraduate) and people at a disadvantage, which may lead to insufficient perspective-taking. Gerace et al. (2015) found a positive relationship between perceptive-taking and past experiences, in that people with similar past experiences could more easily and accurately take the other person’s perspective. Moreover, research shows that undergraduates tend to have limited experiences of negative long-term situations, such as prolonged illness, economic disadvantages, and a lack of family (Case et al. 2004; Kong et al. 2014; Moriña 2017), albeit they all have childhood-related experiences. Accordingly, they may more easily tell the developmental differences between children and adults but may face hindrances when taking into the perspectives of people with prolonged illnesses, orphans, and those economically disadvantaged. Moreover, undergraduate students in China use more internal than external attribution (Yu et al. 2005). Hence, the underestimation of the environment as a factor in creativity development may result in the neglect of the creator’s situation. The disregard for a creator’s prolonged illness, economic disadvantages, and the lack of family also revealed that people use a product-oriented evaluation rather than a creativity–development-oriented evaluation in these conditions. 

Cognitive empathy predicted creative evaluation scores when the participants knew that the creator was at a disadvantage and did not when the participants knew that the creator was at an advantage, supporting H2. This result revealed the importance of understanding the creator’s situation in creativity–development-oriented evaluation. Han et al. (2017) demonstrated that taking the creator’s perspective helps the rater adjust their ratings for creative products. Our results further suggested the importance of understanding. Cognitive empathy is the ability to understand another’s situation and emotions (Spaulding 2017). Understanding the situation of disadvantaged groups means noticing the diversity and considerations of justice. People with high cognitive empathy are more sensitive to justice toward others (Decety and Yoder 2016) and show more fairness in distribution tasks (Rochat et al. 2009). Segal (2011) proposed a triangle model of social empathy involving individual empathy, contextual components, and social responsibility. The model emphasizes that the ability to understand people by perceiving their life situations helps people become aware of inequalities and disparities in life, which then enhances personal social responsibility and contributes to social justice. By showing the positive effects of cognitive empathy on the creative evaluation of products from creators at a disadvantage, our results supported this model and calls for more understanding of disadvantaged groups.

Considering our findings that affective empathy did not predict creative evaluation when the participants knew that the creator was at a disadvantage, H3 and H5 were not supported. One possible explanation for this may be the experience of negative emotions when seeing others in distress; people experience both personal distress and sympathy when they see others in distress (Eisenberg et al. 1989). Recent studies have found that social anxiety is associated with affective empathy but not with cognitive empathy or sympathy (Bray et al. 2021). This tendency to experience other’s uncomfortable feelings may result in more personal distress when seeing others in distress, which may then interfere with the sympathy process (Kim and Han 2018). 

Other findings in the current study suggested that the influence of affective empathy in creative evaluation should be explained by the emotions evoked in a given context. First, we found the negative moderation effect of affective empathy in the relationship between cognitive empathy and creative evaluation in the disadvantaged condition. This concurs with the “overarousal hypothesis,” which describes that people who are prone to intense emotions are more likely to experience overarousal (i.e., personal distress, which relates to both less prosocial and more avoidance behavior) than sympathy (Eisenberg and Eggum 2009; Grynberg and López-Pérez 2018). Accordingly, affective empathy may reduce the effect of cognitive empathy under disadvantaged conditions.

Second, our results showed that affective empathy predicted creative evaluation scores when the participants knew that the creator was at an advantage and that cognitive empathy positively moderated this effect. One possible explanation is the experience of positive emotions when seeing others in advantageous situations. Past studies have found that people easily experience positive empathy and a subsequent positive effect after reading textual descriptions of real-life emotional scenes (Kimmig et al. 2021; Telle and Pfister 2016). The study of Mastria et al. (2019) also showed that this positive emotion then associates with higher creative evaluation scores, with their findings depicting that people in positive states gave higher scores to creative products. This effect can also be explained by the “affective maintenance hypothesis,” which proposes that people in positive affect states will show more prosocial behaviors because they need to maintain this positive state (Telle and Pfister 2016). Additionally, our result showing the moderate positive effect of cognitive empathy on the relationship between affective empathy and creative evaluation in the advantaged condition was consistent with the proposals of the typical empathy model (Batson et al. 1997a; Eisenberg et al. 1989; Spaulding 2017; Telle and Pfister 2016). In this model, people need to first understand the other person’s situation to then experience their emotions based on the situation (e.g., in our research, the other’s situation would be personal distress and empathic concern in the disadvantaged condition and positive emotions in the advantaged condition).

No interaction effect was found between the creator’s situation and sympathy, and no triple interaction effect was found between the creator’s situation, sympathy, and cognitive empathy. Thus, H4 and H6 were not supported. One possible explanation was the difference between evaluation and altruism behavior. Batson and Shaw (1991) defined altruism as a motivational state with the ultimate goal of increasing another’s welfare. Hence, the raters with high sympathy in our study may have had insufficient motivation to adjust their evaluation because the task was not significantly related to the creator’s welfare; specifically, we found that the main effect of sympathy almost reached significance (*p* = .065). These results of sympathy’s role suggested that people with high sympathy may have a potential loose standard in creative evaluation. Past research found that agreeableness, a close personality trait to empathy (Graziano et al. 2007), has an unstable positive correlation with creative product ratings (Tan et al. 2015). Despite the theoretical support (i.e., positive attitude toward the creator and loose standards) for the effect of sympathy on creative evaluation, the related effect in this study was insignificant. Hence, researchers should continue to investigate this topic.

## 5. Practical Implications

Our results suggested that it was not easy for people to use the creator-development-oriented evaluation and that empathy was a key influencing factor in raters’ creative evaluation orientation. Researchers have described that a focus on product-oriented evaluation results in the encumbering of the dynamic process of creativity-in-the-making, which was then accompanied by the rater’s underestimation of the creative product and the creator’s efforts (Beghetto and Kaufman 2007; Moran and John-Steiner 2003; Runco 2005). More than that, it negatively impacted the creator–rater system. People with a disadvantage have a vulnerable mental state, so they are more likely to suffer from mood disorders (Williams et al. 2011) and mental illnesses (Lawrence et al. 2013). Hence, an overemphasis on the creative work’s quality and the neglect of their situation can easily lead to experiencing misunderstandings and negative emotions. If the rater makes no effort or fails in solving the cognitive bias between the rater and the creator, this unresolved misunderstanding could then infiltrate their relationship (Rhodes et al. 1994) and inhibit creativity development. To avoid this phenomenon, more efforts are needed to provide a supportive environment for disadvantaged groups. Our results suggested that one possible way is to enhance the rater’s cognitive empathy. 

This study also contributed to the question of how educators can be good estimators of creativity (Corazza 2016). For students’ development, teachers need to use formative evaluation, rather than summative evaluation, in their educational practice (Bennett 2011). This requires them to understand the situation of the students, including their psychological state, development level, and family background. Cooksey et al. (2007) explored the influence of students’ writing context on their teachers’ evaluation. They found that most teachers assessed students’ writing based on task-related contexts (e.g., the information provided before the writing task, student’s past performance history, student’s effort, etc.), and few teachers assessed students’ writing based on student-related context (e.g., family background, student’s physiological state, community context, etc.). Our results suggested that cognitive empathy is a potential supportive factor to the use of formative evaluation since it facilitates taking on the perspective of the creator’s situation.

## 6. Limitations and Future Research

There were several study limitations. First, only three creative product types were used. Although the product types used are widely used in creativity research, this may limit finding generalizability. 

Second, although there were no measures of emotion in this study, we did observe the influence of affective empathy, suggesting that emotion may play a role in creative evaluation. Emotions related to empathy (e.g., compassion, concern, tenderness, etc) are associated with caring for others (Van Lange 2008). Positive and negative emotions also affect creative evaluation (Mastria et al. 2019). Still, studies are needed to examine the effect of state empathy, positive emotion, and negative emotion in creative evaluation in different contexts. 

Third, more methods are needed to induce empathy. Our results showed that a short description of a disadvantaged group is insufficient for most people to adjust their assessment based on the reality of people with prolonged illnesses, orphans, and those at an economic disadvantage. This was consistent with the study by Raviselvam et al. (2016), which describes that being briefed about the situation of a blind person is insufficient for people to assume the perspective of and solve the daily dilemmas of a blind person creatively. Future research should use more methods to allow for perspective-taking, such as displaying the creator’s situation by listening to a broadcasting program (Batson et al. 1997b) or virtual reality (VR) simulation of the creator’s situation (Lam et al. 2020).

## Figures and Tables

**Table 1:** Descriptive statistics for creative product evaluation by condition and the scores for the RIBS, cognitive empathy, affective empathy, and sympathy.

Conditions	*N*	*Rating of Creative*	*RIBS* ^2^	*CE* ^3^	*AE* ^4^	*S* ^5^
Disadvantage	300	0.093 ± 0.75 ^1^	3.26 ± 0.51	3.26 ± 0.52	3.45 ± 0.55	3.39 ± 0.52
Advantage	290	−0.096 ± 0.70	3.23 ± 0.54	3.32 ± 0.54	3.44 ± 0.60	3.40 ± 0.60

^1^ Numbers are represented as means ± standard deviation. Average z-scores including the figure, story, and idea tasks. ^2^ RIBS: Runco Ideational Behavior Scale. ^3^ CE: Cognitive empathy. ^4^ AE: Affective empathy. ^5^ S: Sympathy.

**Table 2:** The effects of the creator’s situation, gender, RIBS, empathy, and their interactions with creative product evaluation.

Variables	*SS*	*df*	*F*	*p*	*η_p_* ^2^
Model	31.328	17	4.303	< .001 ***	.113
Creator’s situation	9.213	1	18.828	< .001 ***	.019
Gender	0.188	1	0.385	.535	<.001
RIBS ^1^	4.309	1	8.807	.003 **	.022
CE ^2^	0.868	1	1.774	.183	.012
AE ^3^	0.205	1	0.419	.517	<.001
S ^4^	1.668	1	3.410	.065	.005
CE × AE	0.266	1	0.544	.461	.001
CE × S	0.491	1	1.003	.317	.001
AE × S	0.995	1	2.033	.154	.011
Creator’s situation × CE	3.954	1	8.080	.005 **	.006
Creator’s situation × AE	3.203	1	6.545	.011 *	.017
Creator’s situation × S	0.199	1	0.406	.524	.001
CE × AE × S	1.457	1	2.978	.085	<.001
Creator’s situation × CE × AE	2.195	1	4.486	.035 *	.025
Creator’s situation × CE × S	1.394	1	2.848	.092	.004
Creator’s situation × AE × S	0.143	1	0.293	.589	.001
Creator’s situation × CE × AE × S	0.579	1	1.183	.277	.002
Residuals	279.893	572			
Total	311.279	590			

Note: ^1^ RIBS: Runco Ideational Behavior Scale. ^2^ CE: Cognitive empathy. ^3^ AE: Affective empathy. ^4^ S: Sympathy. * *p* < .05, ** *p* < .01, *** *p* < .001.

**Table 3:** Descriptive statistics of the average z-score for the figure, story, and idea tasks by the eight creator’s situations.

Variables		*N*	*M* ± *SD*
Age	Child	70	0.418 ± 0.75
	Adult	74	−0.041 ± 0.70
Physical state	Prolonged illness	77	−0.008 ± 0.71
	Healthy	70	−0.183 ± 0.67
Family situation	Orphan	82	−0.007 ± 0.77
	Big family	79	−0.077 ± 0.74
Economic state	Economically disadvantaged	71	−0.002 ± 0.69
	Economically advantaged	67	−0.089 ± 0.71

## Data Availability

Not applicable.

## Appendix A

The introductions of the creator’s situation on the rating page in different conditions are as follow, child: “Wang Ming is nine years old, in third grade this year;” adult: “Wang Ming is 23 years old, and working just over one year;” prolonged illness: “Wang Ming is in frail health, suffering from prolonged illness;” healthy: “Wang Ming is in robust health, exercising frequently;” orphan: “Wang Ming is an orphan, lives helplessly and is friendless;” big family: “Wang Ming has a big family, with many relatives;” economically disadvantaged: “Wang Ming comes from a needy family, suffering from a housing problem;” economically advantaged: “Wang Ming comes from a well-off family, and has nothing to worry about.”)
